# Midwives' and Diabetes Nurses' Experience of Screening and Care of Women with Gestational Diabetes Mellitus: A Qualitative Interview Study

**DOI:** 10.1155/2023/6386581

**Published:** 2023-07-29

**Authors:** Sofia Köpsén, Mikael Lilja, Margareta Hellgren, Jonas Sandlund, Rita Sjöström

**Affiliations:** ^1^Department of Community Medicine and Rehabilitation, Unit of Research, Education and Development-Östersund, Umeå University, Umeå, Sweden; ^2^Department of Public Health and Clinical Medicine, Unit of Research, Education and Development-Östersund, Umeå University, Umeå, Sweden; ^3^The Skaraborg Institute, Sweden. Department of Public Health and Community Medicine/Primary Health Care, The Sahlgrenska Academy at the University of Gothenburg, Gothenburg, Sweden; ^4^Department of Community Medicine and Rehabilitation, Physiotherapy, Umeå University, Umeå, Sweden; ^5^Department of Community Medicine and Rehabilitation, Unit of Research, Education and Development-Östersund, Umeå University, Umeå, Sweden

## Abstract

**Background:**

Gestational diabetes mellitus (GDM) is increasing and is associated with adverse outcomes for both mother and child. The metabolic demands of pregnancy can reveal a predisposition for type 2 diabetes mellitus (T2DM), and women with a history of GDM are more likely to develop T2DM than women with normoglycemic pregnancies.

**Aim:**

The aim of this study was to explore midwives' and diabetes nurses' experience of their role in screening, care, and follow-up of women with gestational diabetes mellitus and, further, to explore their opinions and thoughts about existing routines and guidelines.

**Method:**

Individual interviews were performed with ten diabetes nurses and eight midwives working in primary and special care. Qualitative content analysis was done according to Graneheim and Lundman.

**Results:**

The analysis of the interviews resulted in the overall theme “An act of balance between normalcy and illness, working for motivation with dilemmas throughout the chain of health care.” Difficulties in carrying out the important task of handling GDM while at the same time keeping the pregnancy in focus were central. Women were described as highly motivated to maintain a healthy lifestyle during pregnancy with the baby in mind, but it seemed difficult to maintain this after delivery, and compliance with long-term follow-up with the aim of reducing the risk of T2DM was low. The women came to the first follow-up but did not continue with later contact. This was at a time when the women felt healthy and were focusing on the baby and not themselves. A lack of cooperation and easy access to a dietician and physiotherapist were pointed out as well as a wish for resources such as group activities and multiprofessional teams.

## 1. Introduction

Gestational diabetes mellitus (GDM) is increasing globally and is associated with adverse outcomes for both mother and child, such as greater risk for type 2 diabetes mellitus (T2DM), macrosomia, and pre-eclampsia [1, 2]. Globally, 14% of all pregnant women develop GDM according to a pooled prevalence number [3], and in Sweden, the prevalence was 5.2% in 2020 [4]. The metabolic demands of pregnancy can reveal a predisposition for T2DM, and women with a history of GDM are more likely to develop T2DM than women with normoglycemic pregnancies [5, 6]. A review from 2020 [5] showed a 10-fold higher risk for developing T2DM, and another review about the incidence of T2DM showed an estimated risk of 19.7% after 10 years and nearly 30% after 20 years [6].

As for other types of diabetes, an increased plasma glucose level is used to diagnose GDM [2]. WHO has changed its recommendations and lowered the cutoff for diagnosing GDM [2]. In 2020, seven of Sweden's 21 regions used the lower cutoff [4]. The CDC4G study [7] is in progress in Sweden to further evaluate whether to implement the lower cutoff recommended by WHO. Screening to detect GDM is performed at the maternity health clinic, based on risk factors such as heredity and overweight, but a few regions offer screening to all pregnant women [4]. Recommendations for screening differ between regions in Sweden [4]. When diagnosed, the woman is referred to a diabetes nurse in special care during pregnancy and for follow-up with the diabetes nurse in primary care postpartum. A lower cutoff for GDM diagnosis would result in an increased number of women with GDM, and this will entail demands on health-care organisations and resources. Therefore, it is important to study how health-care professionals today experience their work with this group of women.

Earlier research is limited concerning health-care professionals' experience of treating women with GDM. In interviews with midwives about their experience providing care and counselling to pregnant women with GDM, Persson et al. [8] identified fear of failure as a central factor affecting the midwives' choice of strategies. On top of their ordinary work tasks, the midwives felt that they needed to give specific counselling regarding GDM and to initiate lifestyle changes. Persson et al. reported a need for a more supportive organisation.

Interviews with Swedish midwives about strategies for handling challenging dietary counselling situations presented ambiguous strategies. The information focused on GDM prevention, but extra challenges were raised when counselling women who were obese, on special diets, had eating disorders, or came from different cultures. Those challenging situations demanded other strategies. The researchers concluded that further education towards a more person-centred approach was needed as well as possible cooperation with dieticians [9].

In a Norwegian study, midwives at a special care unit for women with diabetes mellitus were interviewed. They described a conflict owing to lack of time, where medical issues were prioritised, and person-centred care with a midwifery focus was given less space and time [10].

A qualitative study from Denmark interviewed health-care professionals who met women with GDM during and after pregnancy. They found that collaboration and information between the different clinics and different health-care professionals need to be improved. It was unclear who was to be mainly responsible for long-term follow-up work to prevent T2DM. Due to a lack of guidelines and poor knowledge about existing guidelines, the women received very different care [11].

Examples of the few existing studies [8–11] indicate organisational issues and a clinical struggle to handle and balance different situations and work tasks in a tight time schedule. Thus, a need for further research within the area to map and increase understanding/knowledge about work with patients with GDM is necessary, during pregnancy as well as after delivery. In this study, we want to investigate further how consultations take place in different parts of the chain of health care, and during pregnancy as well as after delivery, both from the midwives' perspective and diabetes nurses' perspective. The results of this study can increase knowledge about daily work in the clinic and thus give important information for possible improvements and changes.

## 2. Aim

The aim of this study was to explore midwives' and diabetes nurses' experience of their role in screening, care, and follow-up of women with gestational diabetes mellitus and, further, to explore their opinions and thoughts about existing routines and guidelines.

## 3. Method

### 3.1. Study Design

A qualitative method with an inductive approach was chosen since we wanted to gain deeper insight into participants' personal experiences [12]. Semistructured individual interviews were performed to explore midwives' and diabetes nurses' experience of their role in screening, treating, and following up on women with GDM. Opinions and thoughts about existing guidelines were also explored.

### 3.2. Participants

All midwives and diabetes nurses in primary care and in special care units working with women with GDM in a region in the northern part of Sweden were invited to participate. This study used a purposive sample [12]. Potential participants were contacted through coordinating personnel and/or the responsible chief of the respective professions, and information was distributed among their contacts. Permission to participate in the interviews during working hours was given by the regional management. Ten diabetes nurses (age 27–61 with median age 46.5, work experience 2–15 years with a median of 5.6) and eight midwives (age 34–63 with median age 47.5, work experience 3–33 years with a median of 15.5) chose to participate in individual interviews. The participants were equally spread between rural and central areas in both groups. Two declined participation due to lack of time, one due to a feeling of lack of experience, and four did not reply to our contact.

Information about the study was given orally and in writing, and participants were able to ask questions. Informed consent was signed before entry into the study.

### 3.3. Data Collection

Data were collected through individual interviews conducted by phone or online, depending on the participants' choice. Face-to-face sessions were not possible due to the ongoing pandemic. All interviews were conducted by the first author, S.K., between May and July 2021. A semistructured interview guide with open-ended questions was used. A pilot interview was performed, and only minor adjustments to the interview guide were made; thus, pilot data were included in the study. The interviews were recorded digitally (Olympus VN-541 PC) and lasted between 12 and 28 minutes with a mean of 20 minutes. There was no time restriction, and the participants were asked at the end whether they had anything to add. Participants agreed to be contacted again later should any questions come up during the analysis, but no such need occurred. Transcripts were not returned to the participants for comments.

### 3.4. Data Analysis

The interviews were recorded digitally, transcribed verbatim by S.K., and deidentified and analysed using qualitative content analysis according to Graneheim and Lundman [13–15]. Qualitative content analysis emphasises variation, through similarities and differences in the material [13–15].

The analysis started with repeated readings of the material to get a sense of its entirety and content. Meaning units were identified and condensed to shorten their content, while preserving their core content, and then, they were labelled with codes to capture their essence. The codes were then grouped and abstracted into categories and subcategories by their commonalities on a manifest level. Further interpretation and abstraction resulted in a theme that exposes the latent content in the material. To ensure dependability, S.K. and R.S. read the material, identified meaning units and coded the material separately, and then compared and discussed the differences until a consensus was reached. Cocreation in the analysis is often described as consensus [14]. Codes were then grouped into categories and subcategories separately, compared and discussed, and adjusted separately and then discussed again until reaching consensus. To further ensure dependability, this material was discussed in the whole group who had read parts of the material. An overall theme was created after discussions. Quotations were chosen to exemplify and clarify. All steps of the analysis process were managed manually. An example of the analysis is shown in Table 1.

### 3.5. Ethics

Ethical approval for the study was obtained from the Swedish Ethical Review Authority, Dnr 2021-00179. All participants gave signed informed consent before the start of the study. Data materials were kept in computer files behind two-factor authentication, and transcripts were kept in a locked cabinet.

## 4. Results

The analysis resulted in four categories, nine subcategories, and one theme (Table 2).

From the categories to subcategories, the theme *“An act of balance between normalcy and illness, working for motivation with dilemmas throughout the chain of health care”* was abstracted and interpreted. The theme originates from the participants' stories about difficulties providing necessary information about the patients' illness while maintaining focus on positive aspects of this special time during pregnancy. They also spoke about difficulties later, at follow-up in addressing the risk of future illness and health risks at a time when the woman is “healthy.” This was a challenge at all parts of the chain of health care, and being supportive and encouraging was regarded as a central issue. The participants expressed different motivational challenges depending on occupation and place in the chain of care.

### 4.1. Structure within and between Caregivers

Different ways of cooperation between caregivers and parts of the chain of health care were described, but so were a lack of cooperation and uncertainty about details in routines and guidelines. Participants described flexibility in the care and contact and possibility to adapt to the patient as important.

#### 4.1.1. Caregivers' Role and Cooperation throughout the Chain of Health Care around the Women

The participants described their role in the care of the women, what their work assignments were, how they referred patients to other caregivers, and/or how patients were referred to them by others. They further reported on different forms of contact, for example, in person, by phone or online, according to the needs and preferences of the individual women. Midwives in primary care felt that their role was central during pregnancy but that their part in considering GDM was small; they set the diagnosis and then referred to “professionals” at the diabetes clinic.

Well, if we detect GDM we refer, we get professional help. We contact the diabetes clinic and then they start doing controls. The women continue with us according to the base programme. You are a key contact from the start to catch and find, and then refer… #13

All women diagnosed with GDM were referred to diabetes nurses in special care for regular contact during pregnancy. However, diabetes nurses in primary care are responsible for the follow-up after delivery (up to a year after delivery), a time when many women consider themselves healthy; the GDM is in the past.

Good experiences of quick nurturing after set diagnosis were common and well spread in the interviews, as were accounts of easy contact between primary care and special care nurses and then prompt contact with the woman after diagnosis. Due to long distances in parts of the area where the interviews were conducted, special care and primary care cooperate in special cases, for example, by having personal meetings with primary care close to home instead of travelling long distances for each visit or having contact by phone.

In primary care, the diabetes nurses felt alone with these patients and had no active cooperation with other caregivers at the clinic.

Some of the midwives communicated to the diabetes nurse at their clinic when a woman was diagnosed with GDM, but this was an individual initiative and not routine. A recurring opinion was that there was no access to a dietician and that this category of competence would be important and beneficial in the work with those women.

Also, cooperation with a physiotherapist was lacking, as were group activities, which many participants thought would be beneficial for this patient group. The opportunity to meet others in the same situation and support each other was thought to be very rewarding.

#### 4.1.2. Thoughts about Routines and Guidelines

Even within the same profession, there were different opinions about existing routines and guidelines. Some thought they were clear and easy to follow, while others saw a need for clarification and improvement. It was apparent that there were some details that needed clarification, based on diverse stories about how, for example, referrals should be made.

Our routines are a bit fuzzy when it comes to screening for GDM. A bit vague. I have pointed that out before; maybe it will change… #17

Some differences were seen in the layout of the work, and several participants expressed a wish for clearer guidelines to ensure equal care. Some had developed local guidelines on how to perform follow-ups at their clinic to provide clarity when the regional guidelines were considered unclear and/or not updated.

Different tools and resources were used to accomplish the work tasks. Some used the diabetes handbook available online [16] along with other patient information also available online. The women were, therefore, getting more diverse information than they might have if there had been more detailed regional guidelines.

A recurrent notion that came up in the interviews was that some women might be missed and that the routine of referring women with previous GDM to a special care diabetes nurse directly at the enrolment meeting with the midwife needed clarification. The participants also indicated uncertainty about glucose tolerance tests and said there was a need to clarify the routine and follow-up requirements. Another lack in the routines that came up was that the diabetes nurses in special care do not have access to the medical journals of midwives in primary care, which may impede the communication between caregivers.

There were questions and uncertainty about potential new cutoff limits for the diagnosis of GDM in the future. This raised thoughts about a possible need for a change in organisational responsibilities due to a growing number of patients.

### 4.2. Content of the Daily Tasks

Different work tasks were described in the interviews. All caregivers focused on a healthy lifestyle as well as screening and follow-up.

#### 4.2.1. Screening and Sampling

Collecting and handling blood samples was described as an important part of the work tasks, first to diagnose GDM and then to follow blood glucose levels over time during pregnancy as well as during long-term follow-up after delivery. Screening and sampling were seen as essential in order to detect GDM and prevent further illness. Screening is done based on risk factors and is important for detecting GDM, but several midwives said that many cases are found by the random testing of blood glucose that is performed during pregnancy.

It can be during our consultations that blood glucose shows that you need to progress to do an OGTT or you do it due to previous risk factors. And a GDM turns up. #4

After diagnosis, the women with GDM are taught to self-monitor blood glucose levels regularly and report to their diabetes nurse at the special care unit. After delivery, the routine is to offer recurrent follow-ups with the diabetes nurse in primary care, and blood samples are also an important part of this.

#### 4.2.2. Promote a Healthy Lifestyle and Encourage the Normal

All participants saw the provision of lifestyle information as a central task in their daily work and talked about how to promote a healthy lifestyle, saying that this should be presented as the normal way of living and taking care of yourself. Healthy eating and physical activity were viewed as the main objectives.

In general we inform everybody about lifestyle factors. When you are pregnant, you are prone to making lifestyle changes, this we know. Many start exercising, start to be aware of weight and not gaining too much, thinking about what they are eating. Many think about what they are eating…. It's quite general, regardless of whether you are a diabetic or not… #8

For the midwives in primary care, despite the GDM, the emphasis was on keeping the pregnancy and baby in focus and giving the mother-baby connection priority.

Providing support and reassurance while giving women the opportunity to ask questions was important in getting the information out effectively. The desire to do everything possible to ensure the baby's well-being during pregnancy was perceived as a key motivator for the women to adopt lifestyle improvements before childbirth. Later, after childbirth, this could be more difficult since the focus shifts to the baby and family and is not so much on the mothers themselves. Many spoke of the importance of having healthy habits for the future and indicated that further support is needed to help mothers maintain a healthy lifestyle.

#### 4.2.3. Inform and Increase Knowledge of Risks and Consequences

Another important work task the participants described was providing information and increasing knowledge about diabetes and its risks and consequences. The importance of continuing this work after delivery, when most women consider themselves healthy, was seen as a key issue.

I think it is very important to follow up. Since they have such a big risk of developing T2DM… Yes, it is important to follow up and to inform them. #5

The midwives perceived that increasing body mass index prepregnancy is getting more prevalent, and mental health issues are more common. This makes the work, and helping mothers become role models for their children even more important.

#### 4.2.4. Compliance and Course of Action at Follow-Up

The participants expressed that compliance was generally good during pregnancy, but it became more difficult with the follow-ups after delivery. Many participants described a long time delay between delivery and the first follow-up. They found that the women would come to the first visit for follow-up but would not continue with regular appointments in the long run, since they were feeling healthy at this point and were focused on the baby and the family. Attempts had been made to customise follow-up routines according to individuals' needs and preferences, but the compliance was still low.

I think it is easy as a patient to think that I have good blood sugar and that there is nothing to worry about now. But you still have to make them understand that the risk of developing T2DM is quite big. #6

The informants described uncertainties and differences concerning strategies and the interval when it came to follow-ups in primary care after delivery. The diabetes nurses expressed that, in the end, it came down to the woman's own responsibility. The low compliance for long-term follow-up was considered problematic.

### 4.3. Complexity of Counselling Situations

Obstacles, challenges, and difficulties of course occur in the contacts with these patients. Motivational work, normal impacts of pregnancy, and cultural differences, including language barriers, were the main subjects spoken about.

#### 4.3.1. Challenges in the Daily Work

Different challenges were reported in the interviews. A recurrent topic was difficulties concerning motivation. Participants indicated that health education and motivation were time-consuming and that time was lacking. The work of changing habits and lifestyles is also time-consuming, so the same problem with lack of time is connected to this. Anxiety was quite common among the mothers-to-be, with different levels of worry among the women, both for the coming babies and for themselves. Anxiety could be a difficulty and an impediment, but it could also motivate women to change and to maintain new habits. Many participants reported that motivation was higher during pregnancy than after delivery.

To keep them motivated, it is different while they are pregnant; then they have someone else to think about./… ./It is the same as that you transmit good lifestyle to the children, that is what I think about, teaching the children to be physically active along with eating healthy. #9

It was a big challenge to try to keep a recurrent contact over time after delivery. Maintaining healthy eating and physical activity was considered a challenge. Another challenge was complex counselling situations on delicate matters such as weight and being monitored with a scale, which was very sensitive for many of the women. The participants felt that they could do better if they had more time and resources.

#### 4.3.2. Obstructive Physical Impact of Pregnancy

A recurrent topic in the interviews concerned the physical strains and events that normally occur during pregnancy that could be a complicating factor for lifestyle changes. For example, nausea and cravings make it even harder to keep a healthy diet, and pregnancy-related lumbopelvic pain can make physical activity harder. Even during a healthy pregnancy, there are many emotions and bodily events to manage.

To encourage them to exercise. Sometimes they get stuck in that they cannot walk because of pain from joint loosening in the pelvis and all that. So there is that… To get them more physically active early. #10

#### 4.3.3. Language and Cultural Differences

Many of the women the participants meet are immigrants, and this often raises difficulties related to language and communication as well as cultural differences such as different food cultures and customs when it came to physical activity.

Dietary habits and general… this with cultural differences… You can notice big differences in what people eat and that there are different cultures. #4

Communication via interpreter was described as an aggravating factor in the consultation situations that made it more difficult to connect with the mother and to ensure that all the vital details in the conversation were clearly relayed.

Moreover, some women are illiterate and therefore cannot assimilate written information indicating, for example, the carbohydrate content in various products or other written information which would complement and repeat oral information. In addition, the numbers displayed on the blood sugar tester might not be understood by women in this group.

When you don't speak the same language and perhaps need an interpreter. And the interpretation does not always work well. Now during COVID-19 there have only been telephone interpreters, and you do not always reach all the way. Then there are many who can't read or count; if the woman is illiterate, that is very difficult. #12

### 4.4. Professional Competence and Need for Further Education

The participants had a varying number of years in the profession and the desire for further training varied, not always connected to longer or shorter experience. Many wanted further education to be given the opportunity and time to learn about new findings and new knowledge within the area.

I would be grateful for more education since this… I meet so few patients that I feel I don't… I don't work with it so much that I feel so very secure in what I do. The more you meet a group of patients the more secure you get within that area. And there are not that many patients, so it would have been nice to get some updates and more education. #16

A recurrent wish was to have the opportunity to learn more about areas that were not their speciality, for example, more knowledge about pregnancy for the diabetes nurses and so on. This would enable them to answer questions from the women, but they also wanted the information to increase their own understanding. A need for further education about cultural differences and, for example, food cultures was expressed as well as further training in conversation methodology.

## 5. Discussion

### 5.1. Result Discussion

This study aimed to explore midwives' and diabetes nurses' experiences of screening and care of women with GDM during and after pregnancy. The participants were active in different phases, according to their profession and placement in the chain of health care. Descriptions of the act of balancing between normalcy and illness came up several times in the interviews, and this has also been reported in previous research concerning health-care professionals' experience working with women with GDM [8, 10]. This balancing act was described in different ways; for example, that a lack of time puts the illness in priority instead of the pregnancy and also that it was important to emphasise that lifestyle advice should be considered for everybody, thus as something normal and not specifically because of their GDM. The informants thought this was an important group of patients that need time and care, and also that it was important to consider that the health of the mother would affect the health of the baby and family for a long time to come.

It was noticeable in the material that there were different opinions about routines and guidelines among the informants and that there were some misunderstandings about who does what and thinking that certain tasks were someone else's responsibility. There was a lack of knowledge and understanding of how work tasks and information were organised and distributed between professions and between parts of the chain of health care. Similar difficulties were pointed out in a Danish study [11], where suggestions about an overview of organisation, collaboration, and information transfer were made. A study from 2009 [17] showed low compliance with guidelines about risk factor-based screening. Only 30.7% of women with one or more risk factors were exposed to OGTT.

Different primary health-care centres had developed their own, more specific, routines, which caused diversity between units. This was time-consuming work that, in a situation with well-functioning routines, should not have been necessary, and it led to a risk of unequal care. A related issue that should be possible to solve more effectively was that most participants found written information online to hand out to their patients since no such things were in the local guidelines. Of course, this provided opportunities to adapt the information to individual women, taking into consideration language and depth of information, but it could also be time-consuming and lead to unequal care. Besides the local guidelines, there are also national guidelines published by the National Board of Health and Welfare (Socialstyrelsen) concerning the prevention and treatment of unhealthy lifestyle habits. These guidelines can be of some help in ensuring the provision of equal treatment and care. The national guidelines stipulate person-centred care adapted to the individual, with a focus on patient education and support [18]. This was a focus that the informants spoke about frequently.

Cooperation concerning referrals from primary care midwives who had diagnosed women with GDM to diabetes nurses in special care seemed to be fast and effective, although there were some uncertainties about the correct way to make a referral. Beyond this, there was very little cooperation between special care and primary care and between occupational groups within primary care. Several participants remarked on the lack of a dietician and expressed a wish for further cooperation with a physiotherapist. It would be beneficial to have access to a multiprofessional team [19].

In both special and primary care, participants expressed a wish to be able to offer different group activities/treatments in order to take advantage of the strength in a group, where patients can share experiences and support each other. Previous research has shown the benefits of groups, which can improve maintenance and adherence [20]. Identification has been pointed out as an important factor when attending a group treatment, and a facilitator is needed for successful treatment [21, 22]. A review shows the effect of social identification-building on health, offering the possibility to identify with and belong to a group [21].

A clear organisational detail that would simplify and secure the work for diabetes nurses in special care would be to enable them to read midwives' reports from the women's primary care appointments. This was not possible at the time of the interviews.

A key challenge was communication since many women are immigrants and have no or only a limited ability to communicate in the Swedish language. Communication through professional interpreters or with the help of a relative is a complicating factor in the personal meeting and makes it more difficult to create a relationship and to empower the woman [23]. Furthermore, immigrants may bring different cultural customs when it comes to food and physical activity and different opinions about how women should take care of themselves during pregnancy [23, 24]. Transcultural understanding and competence are important, and health-care professionals need to get proper education in this to be able to meet those patients [24, 25]. Previous research also points out the importance of education and competence on how to collaborate with an interpreter, which is vital for a successful communication [24, 26]. The diabetes nurses also talked about challenges concerning women who are illiterate and who therefore have difficulties understanding what the numbers on the blood glucose tester mean. This leads to difficulties in getting the daily feedback that those measures are supposed to provide. The inability to interpret those results leads to the need for resource-intensive recurrent personal meetings that would otherwise often be managed by phone or digital messages. It has been previously pointed out that illiteracy is a complicating factor that adds to the workload, and that cultural differences create a need for information that is adapted to be made more broadly accessible [27].

The diabetes nurses in primary care felt alone with those patients, women previously diagnosed with GDM, and came in contact with the women quite late after delivery, without having had former contact or any relationship with them. A known facilitator for compliance with recommendations given at consultations is connection and continuity with the caregiver, and this could be a factor here [28, 29]. The compliance following such consultations was experienced to be low, which has also been highlighted in previous studies [29–31]. Often the woman came to the first consultation after delivery but then as time passed, the attendance rate decreased. These consultations are introduced when the woman feels healthy, the baby and the family are in focus, and the risk of T2DM feels distant and no longer relevant. Kim et al. describe how women, despite knowing that GDM gives an increased risk of developing T2DM, did not see themselves as having an increased risk [32]. The consultations are voluntary, and a lot comes down to the woman's own responsibility and interest. Interviews with women treated for GDM report a lack of coordination, unclear responsibility for follow-up among health-care professionals, and absence of individual focus as factors contributing to low compliance with long-term follow-up [29].

If the lower cutoff for diagnosing GDM is implemented, the number of women diagnosed with GDM will increase, leading to new demands on health-care resources and organisations. An organisation where diabetes nurses in primary care have contact with the woman during pregnancy could improve continuity and might improve compliance with long-term follow-up. Continuity in contact and a person-centred approach are facilitating factors [28, 29].

Opinions about more education were varied. Different thoughts about the need and wish for knowledge about the part that was not the focus of one's own profession affected opinions about whether or not more education is needed, for example, for midwives to learn more about diabetes, or for diabetes nurses to know more details about pregnancy and its effects.

### 5.2. Methodological Discussion

The study's aim to explore participants' experiences led to the choice of qualitative method, more specifically, interviews analysed according to Graneheim and Lundman [13–15]. With interviews, there is a chance to go deeper into the subject with supplementary questions, which is not a possibility when using a survey. In this study, semistructured individual interviews were conducted by phone or online. Face-to-face sessions were not possible due to the ongoing COVID-19 pandemic. Bryman [12] discusses the pros and cons and possible differences in results between face-to-face interviews and interviews by phone or other means. There have been concerns that phone interviews might be less rich in content compared to face-to-face sessions, but according to Bryman [12], this has not been shown. By phone, there is a lack of body language, but you can still hear, for example, changes in tone and speed of talk. In an online interview, you can observe body language to some extent. Not conducting interviews face-to-face can be time- and cost-effective and might enable participation despite long distances. The participants in this study could choose which format they considered most convenient, and even over long distances, this made it equally easy for everybody in the area to participate, which is seen as an advantage.

The original plan was to hold focus group interviews, but this was changed to individual interviews due to the pandemic, which prevented in-person meetings. Holding focus group meetings online or by phone was not considered adequate since it would be very difficult to get a good group dynamic and discussion by those means. Focus groups would have been interesting, giving the possibility to observe how individuals act as members of a group discussing a special topic and how they react to others' opinions and experiences [12, 33].

There is no consensus on how many informants are needed in qualitative research and no way to calculate this compared to how participants are calculated in qualitative research. Kvale recommends 5–25 informants, to be able to overview the text material [33]. Too many informants and a big set of data could make it difficult to analyse all data as deeply as needed, while too few or thin materials do not give the necessary richness to the material [12, 33, 34]. The 18 interviews in this study were considered to provide a good amount of data, offering material with richness and to be able to have an overview of the material, analysing similarities and variation.

A purposive sample was used as the researchers turned directly to the group of professionals actively working with this patient category and who thus have knowledge of and experience with the topic [12]. The participants were equally spread between midwives and diabetes nurses, working in rural and central areas, and they were spread among ages and years of work experience. This contributed to the width of the content of the interviews, which is important for the credibility as well as transferability of the study [13, 35].

The use of a semistructured interview guide ensured similar questions and topics in all interviews while still maintaining a flexible interview process. The questions do not have to be asked in the same order to all informants, which gives space and possibility for the informants' opinions and wishes to share [12]. The questions were not distributed beforehand so there was no chance to prepare specifically, but knowing the purpose of the interview made it possible to prepare more generally and consider what they wanted to share. This gives spontaneous answers, which are considered preferable. A pilot interview was conducted to test the interview guide and to make sure that it gave rich answers correlating with the aim of the study. This strengthens the study's credibility [35].

The analysis was done manually, without software. As described in the Method section, the analysis was performed in specific steps and with discussions, cooperation, and consensus between the authors. No member check was performed, since there were no specific questions and because this type of control is criticised [12]. The inclusion of more than one researcher in the analysis strengthens dependability [13, 35]. Furthermore, the process—from recruiting participants, through the analysis and production of finished result—is thoroughly described, which enables the reader to follow along and thereby to judge the study's transferability and credibility. Different steps and examples of the analysis are presented, and the results are clarified in the text and confirmed with quotations, which further strengthens dependability as well as transferability [13, 35]. Quotations help to show that the text in the results comes from the collected data, strengthening conformability as well as credibility [35].

As described in the Method section, the research group was involved in the analysis.

An interview is considered to be a cocreation between the interviewer and the interviewee and later between researcher and text through interaction in the situation [14, 33]. This makes it important for the researchers to be aware of preunderstanding that might affect the process. The authors have strived to be objective. The interviewer had no previous experience working with GDM and does not have the same occupation as the informants.

## 6. Conclusion

Our results demonstrate how women with GDM postpartum, with normalised glucose parameters and a new life situation, often have problems holding on to a healthy lifestyle and that they are often lost to follow-up from the health care. The participants describe that their care organisations have a lack of structure, coordination, and sometimes the knowledge to meet the specific needs linked to follow-up after GDM aiming at long time follow-up, health promotion, and diabetes prevention. If the lower cutoff recommendations from WHO for GDM would be implemented in Sweden, with an increasing number of GDM diagnoses, the urge for organisational changes will be even more relevant.

## Figures and Tables

**Table 1:** Example of the analysis.

Meaning unit	Condensed meaning unit	Code	Category	Subcategory
It's not only, I meet other patients with diabetes with foreign background, and there are difficulties with the language… Then it's good that you can take information from the Internet in their own language	Meet other with diabetes with foreign background. Language difficulties. Good to have information on the Internet in their language	Language difficulties Information in native language	Complexity of counselling situation	Languageand cultural differences

**Table 2:** The results of the analysis.

Category	Subcategory	Theme
Structure within and between caregivers	Caregivers' role and cooperation throughout the chain of health care	
	Thoughts about routines and guidelines	

Content of the daily tasks	Screening and testing/sampling	An act of balance between normalcy and illness, working for motivation with dilemmas throughout the chain of health care
	Promote healthy lifestyle and encourage the normal	
	Inform and increase knowledge of risks and consequences	
	Compliance and course of action at follow-up	

Complexity of counselling situations	Challenges in the daily work	
	Obstructive physical impact of pregnancy	
	Language and cultural differences	

Professional competence and need for further education		

## Data Availability

The data supporting the current study are available from the corresponding author upon request.
